# Diagnostic accuracy of venous manometry to predict elevated intracranial pressure

**DOI:** 10.3389/fneur.2026.1753428

**Published:** 2026-02-10

**Authors:** Timothy White, Kevin Shah, Brendan Ryu, Shyle Mehta, Justin Turpin, Jared Bassett, Brianna Donnelly, Kadir Ozler, Miriam Shao, Cassidy Werner, Kyriakos Papadimitriou, Golnaz Moazami, Jamie Mitchell, Robert Rothstein, Howard Pomeranz, Athos Patsalides

**Affiliations:** 1 Donald and Barbara Zucker School of Medicine, Hofstra University, Hempstead, NY, United States; 2 New York Eye and Ear Infirmary of Mount Sinai, New York, NY, United States

**Keywords:** idiopathic intracranial hypertension (IIH), intracranial pressure (ICP), lumbar puncture (LP), venous manometry, venous sinus stenosis, venous sinus stenting (VSS)

## Abstract

**Background and purpose:**

Venous sinus stenting (VSS) is a well-established treatment for idiopathic intracranial hypertension (IIH). Diagnostic workup includes lumbar puncture (LP) for intracranial pressure (ICP) measurement and catheter venography with manometry to assess venous sinus stenosis. We evaluated the diagnostic accuracy of venous manometry in predicting elevated ICP.

**Materials and methods:**

We retrospectively reviewed patients with venous sinus stenosis who underwent catheter cerebral venography with venous pressure (mmHg) recording and fluoroscopy-guided LP with cerebrospinal fluid opening pressure (CSF-OP, cmH₂O) measurement during the same session. Elevated ICP was defined as CSF-OP ≥20 cmH₂O. Linear regression assessed relationships between venous pressures, trans-stenotic gradient (TSG), and CSF-OP. Sensitivity, specificity, and receiver operating characteristic (ROC) analyses were performed.

**Results:**

84 female patients (mean age 35.5 years) with ≥50% stenosis of the dominant or codominant lateral venous sinuses were included. TSG demonstrated the strongest correlation with CSF-OP (adjusted R^2^ = 0.57, *p* < 0.05), followed by superior sagittal sinus (SSS) pressure (adjusted R^2^ = 0.53, *p* < 0.05). ROC analysis showed areas under the curve of 0.87 for TSG, 0.84 for SSS pressure, and 0.94 when combined. Optimal thresholds were SSS pressure ≥15 mmHg (sensitivity 0.88, specificity 0.68) and TSG ≥ 6 mmHg (sensitivity 0.86, specificity 0.80). In patients with TSG ≥ 6 mmHg, an SSS threshold of 15 mmHg yielded sensitivity 0.94 and specificity 0.85.

**Conclusion:**

SSS pressure and TSG strongly predict ICP elevation in venous sinus stenosis, with combined thresholds providing the highest diagnostic accuracy.

## Introduction

Idiopathic intracranial hypertension (IIH) is a life-altering neurological disorder characterized by elevated intracranial pressure (ICP) (1). With the rising global prevalence of obesity, IIH has become an increasingly important condition requiring timely diagnosis, treatment, and follow-up (2). The diagnostic criteria for IIH—including elevated cerebrospinal fluid opening pressure (CSF-OP), papilledema, and characteristic neuroimaging findings—have evolved since Walter Dandy’s original description in 1937 (2, 3).

The introduction of venous sinus stenting (VSS) has marked a significant advancement in the understanding and management of IIH (4–7). VSS has demonstrated significant efficacy in appropriately selected patients, often resulting in rapid and sustained clinical improvement (6, 8). Studies of the cerebral venous system suggest that IIH may be closely associated with venous sinus stenosis and impaired cerebral venous outflow (9, 10). Whether venous sinus stenosis represents a primary obstruction of venous outflow or a secondary collapse caused by elevated ICP remains an area of active debate. Animal models and human studies have demonstrated a strong association between elevated ICP and increased venous sinus pressures (11–14). Elevated venous pressures may, in turn, reduce cerebrospinal fluid (CSF) absorption, potentially impairing glymphatic flow and exacerbating intracranial hypertension (7).

VSS is generally considered effective only when significant flow-limiting venous sinus stenosis is present; therefore, an extensive clinical evaluation is required before proceeding with intervention. Candidates for VSS typically undergo a lumbar puncture (LP) to confirm elevated ICP, along with catheter venography and manometry to assess the trans-stenotic pressure gradient (TSG) and confirm the presence of hemodynamically significant venous sinus stenosis. Although these criteria are widely used to select VSS candidates, there are limited data directly correlating cerebral venous pressure (CVP) with ICP as measured by CSF-OP. At our institution, CVP and CSF-OP are routinely measured during the same procedural session. Given the proposed pathophysiologic relationship between CVP and ICP, we sought to evaluate the diagnostic accuracy of venous manometry in predicting elevated CSF-OP in patients with venous sinus stenosis. The objective of this study was to determine which venous manometry parameters most accurately predict elevated CSF-OP obtained during the same session. Clarifying this relationship may help refine diagnostic pathways and improve patient selection for VSS.

## Materials and methods

### Patient selection

Patients with symptoms consistent with intracranial hypertension and contrast-enhanced MR venography findings concerning for venous sinus stenosis were referred to the principal investigator by neurologists. All referred patients underwent a standardized diagnostic evaluation, including assessment by neuro-ophthalmology to evaluate for papilledema. Patients were initially treated with a trial of acetazolamide. Awake diagnostic catheter venography with venous manometry—performed during the same session as LP—was obtained only in patients who failed medical therapy and whose symptoms (e.g., papilledema, headache, cognitive complaints, pulsatile tinnitus) remained significant. Performing LP during the same session as manometry allowed for reassessment of CSF-OP under consistent physiologic conditions and provided updated ICP measurements to guide decisions regarding VSS. Not all patients carried a formal diagnosis of IIH at the time of referral; rather, all patients were evaluated for possible IIH associated with venous sinus stenosis based on symptoms and imaging findings. Accordingly, some patients were expected to have normal CSF-OP despite undergoing venous manometry and LP.

Following institutional review board approval with a waiver of consent, we conducted a retrospective review of all consecutive patients who underwent catheter cerebral venography with manometry (mmHg) and a fluoroscopy-guided LP to record CSF-OP (cmH_2_O) during the same neurointerventional procedure between 2020 and 2022. Eligible patients had symptoms consistent with intracranial hypertension and ≥50% stenosis of the dominant lateral venous sinus (or bilateral stenosis if the sinuses were codominant) on contrast-enhanced MR venography.

Collected data included demographics, superior sagittal sinus (SSS) pressure, proximal and distal transverse sinus pressures, proximal and distal sigmoid sinus pressures, jugular bulb pressure, CSF-OP, dominant venous drainage side, and whether patients subsequently underwent VSS. The trans-stenotic pressure gradient (TSG) was calculated as the proximal transverse sinus pressure minus the distal sigmoid sinus pressure.

### Procedure details

Venography, venous manometry, and LP were performed during the same procedure. Systemic sedation was minimized to maintain patient wakefulness and ensure accurate venous pressure measurements. Local anesthesia with lidocaine was used, and systemic sedatives—administered at the discretion of anesthesia—typically consisted of small doses of propofol, dexmedetomidine, midazolam, or fentanyl. Patients were off ICP-lowering medications (e.g., acetazolamide, topiramate) for several weeks prior to the procedure to allow accurate assessment of venous manometry and CSF-OP. Venography was performed before LP to avoid pressure alterations related to CSF drainage.

A 5 Fr sheath was introduced into the right femoral vein, and a 5 Fr Envoy guide catheter was advanced into the internal jugular vein (IJV) on the side of dominant venous drainage, corresponding to the stenotic transverse sinus identified on MR venography. A Headway 27 microcatheter was navigated into the mid SSS, connected to a pressure monitoring system zeroed at the mid-axillary line, and used to record venous pressures. Sequential pressure measurements were then obtained as the microcatheter was withdrawn through the proximal and distal transverse sinus, sigmoid sinus, jugular bulb, and IJV. All measurements were obtained through a single, unilateral femoral venous access. Upon completion, all catheters were removed, and hemostasis was achieved with manual compression and a 5 Fr Vascade closure device.

Patients were then positioned in the left lateral decubitus position. LP was performed under fluoroscopic guidance at the L3–4 or L4–5 level using a 22-gauge needle. CSF-OP was measured with a manometer prior to CSF release.

### Statistical analysis

CSF-OP measured by LP was used as the reference standard, with values ≥20 cmH₂O considered elevated, consistent with accepted high-normal thresholds in adults (15, 16). Linear regression analyses were performed between venous pressures at various sites, TSG, and corresponding CSF-OP values. The strongest correlations were observed for SSS pressure and TSG, which were then selected for further diagnostic accuracy analysis.

Sensitivity and specificity were calculated using the CSF-OP cutoff of ≥20 cmH₂O. The Youden index was computed to identify optimal thresholds for venous pressure measurements, and receiver operating characteristic (ROC) curves were generated. Additional sensitivity and specificity analyses were performed in patients with TSG ≥ 6 mmHg and across a range of SSS pressure thresholds.

## Results

### Patient characteristics

84 patients were included in the final analysis. All were female, with a mean age of 35.5 years and a mean BMI of 33.4 kg/m^2^ (Table 1). The right and left lateral venous sinuses were dominant in 76.2 and 15.5% of patients, respectively, while 8.3% had codominant sinuses. The mean CSF-OP was 25.8 cmH₂O (SD 10.3). The mean SSS pressure was 20.9 mmHg (SD 10.2), and the mean TSG was 10.4 mmHg. Most patients (49/84) had a TSG ≥ 8 mmHg. 35% of patients underwent VSS.

**Table 1:** Patient characteristics.

Variable	Value
Age (years)	Mean: 35.5
Sex, *n* (% female)	84 (100%)
BMI (kg/m^2^)	Mean: 33.4
Dominance (%)	
Right	76.2
Left	15.5
Codominant	8.3
CSF-OP (cmH_2_O)	Mean: 25.8 ± 10.3
SSS Pressure (mmHg)	Mean: 20.9 ± 10.2
TSG (mmHg)	Mean: 10.4
VSS Performed, *n* (%)	29 (35%)

BMI, body mass index; CSF-OP, cerebrospinal fluid opening pressure; SSS, superior sagittal sinus; TSG, trans-stenotic gradient; VSS, venous sinus stenting.

### Linear regression results

TSG demonstrated the highest adjusted R^2^ (0.57, *p* < 0.05) with the lowest residual standard error (6.7), indicating a stronger correlation with CSF-OP compared with other venous pressure measurements (Table 2). SSS pressure demonstrated the second strongest association, with an adjusted R^2^ of 0.53 (*p* < 0.05) and a residual standard error of 7.1. Both TSG and SSS pressure outperformed other measurements in explaining the variance in CSF-OP (Table 2). Based on these results, TSG and SSS pressure were selected for receiver operating characteristic (ROC) analysis. A combined threshold incorporating TSG ≥ 6 mmHg and varying SSS cutoffs was also evaluated to improve diagnostic accuracy.

**Table 2:** Linear regression performance of venous pressure measurements for predicting cerebrospinal fluid opening pressure.

	TSG	GVG	SSS	PTS	DTS	PSS	DSS	JB
F-statistic	111.90	77.42	91.39	87.95	71.44	3.62	2.22	2.84
R	0.76	0.70	0.73	0.72	0.68	0.21	0.16	0.19
R-squared	0.58	0.49	0.53	0.52	0.47	0.04	0.03	0.04
Adjusted R-squared	0.57	0.49	0.53	0.51	0.46	0.03	0.01	0.02
*p*-value	0.00	0.00	0.00	0.00	0.00	0.06	0.14	0.10

TSG, trans-stenotic gradient; GVG, global venous gradient (from superior sagittal sinus to jugular bulb); SSS, superior sagittal sinus; PTS, proximal transverse sinus; DTS, distal transverse sinus; PSS, proximal sigmoid sinus; DSS, distal sigmoid sinus; JB, jugular bulb.

### Comparison of CSF-OP and SSS pressure

Within-patient paired comparison demonstrated that CSF-OP exceeded SSS pressure in 82.9% of patients, whereas SSS pressure exceeded CSF-OP in 19.5% of patients. The signed difference (CSF-OP − SSS pressure) had a mean of 4.7 cm H₂O and a median of 3.5 cm H₂O. Absolute differences demonstrated substantial inter-individual variability, with a mean of 6.74 ± 7.53 cm H₂O, a median of 5 cm H₂O, and a range of 0–25 cm H₂O. These findings indicate that CSF-OP and SSS pressure are not interchangeable and do not demonstrate a consistent 1:1 relationship at the individual patient level.

### Diagnostic accuracy of venous pressure to predict elevated opening pressure

**Figure 1:**
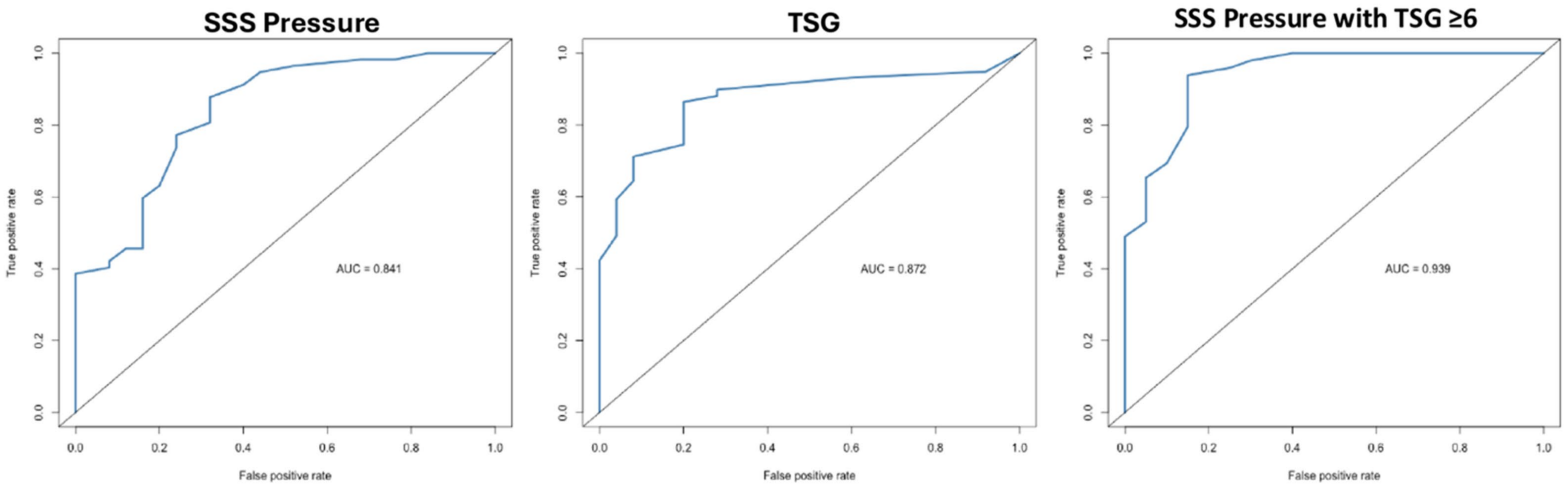
Receiver operating characteristic curves for venous manometry parameters predicting elevated cerebrospinal fluid opening pressure (lumbar puncture ≥20 cmH₂O). SSS, superior sagittal sinus; TSG, trans-stenotic gradient.

The area under the curve (AUC) for SSS pressure in predicting CSF-OP ≥20 cmH₂O was 0.84, compared with 0.87 for TSG and 0.94 for the combination of SSS pressure with TSG ≥ 6 (Figure 1). Youden’s index analysis identified an optimal SSS threshold of 15 mmHg, yielding a sensitivity of 0.88 and specificity of 0.68 (highlighted in Table 3). For TSG, the optimal threshold was 6 mmHg, with a sensitivity of 0.86 and specificity of 0.80 (highlighted in Table 3). Among patients with TSG ≥ 6, the optimal SSS cutoff remained 15 mmHg, improving sensitivity to 0.94 and specificity to 0.85 (highlighted in Table 3). Additional significant thresholds included an SSS pressure ≥27 mmHg and a TSG ≥ 15, both of which were 100% specific for CSF-OP ≥20 cmH₂O. A complete summary of sensitivity and specificity values is provided in Table 3.

**Table 3:** Sensitivity and specificity of superior sagittal sinus pressure and trans-stenotic gradient for predicting lumbar puncture ≥20 cmH₂O, including Youden’s index–identified optimal cutoffs and additional high-specificity thresholds.

TSG				SSS				SSS + TSG ≥ 6			
Gradient	Sensitivity	Specificity	Youden Index	Pressure	Sensitivity	Specificity	Youden’s Index	SSS Pressure	Sensitivity	Specificity	Youden
37.00	0.02	1.00	0.02	53.00	0.02	1.00	0.02	53.00	0.02	1.00	0.02
29.00	0.03	1.00	0.03	52.00	0.04	1.00	0.04	52.00	0.04	1.00	0.04
28.00	0.05	1.00	0.05	48.00	0.05	1.00	0.05	48.00	0.06	1.00	0.06
26.00	0.07	1.00	0.07	40.00	0.07	1.00	0.07	40.00	0.08	1.00	0.08
24.00	0.08	1.00	0.08	37.00	0.12	1.00	0.12	37.00	0.14	1.00	0.14
23.00	0.10	1.00	0.10	36.00	0.14	1.00	0.14	36.00	0.16	1.00	0.16
22.00	0.12	1.00	0.12	35.00	0.16	1.00	0.16	35.00	0.18	1.00	0.18
21.00	0.14	1.00	0.14	33.00	0.18	1.00	0.18	33.00	0.20	1.00	0.20
20.00	0.17	1.00	0.17	32.00	0.19	1.00	0.19	32.00	0.22	1.00	0.22
19.00	0.29	1.00	0.29	31.00	0.23	1.00	0.23	31.00	0.27	1.00	0.27
18.00	0.32	1.00	0.32	29.00	0.26	1.00	0.26	29.00	0.31	1.00	0.31
17.00	0.39	1.00	0.39	28.00	0.33	1.00	0.33	28.00	0.39	1.00	0.39
16.00	0.41	1.00	0.41	27.00	0.39	1.00	0.39	27.00	0.45	1.00	0.45
15.00	0.42	1.00	0.42	26.00	0.40	0.92	0.32	26.00	0.47	1.00	0.47
14.00	0.49	0.96	0.45	25.00	0.42	0.92	0.34	25.00	0.49	1.00	0.49
12.00	0.51	0.96	0.47	24.00	0.46	0.88	0.34	24.00	0.53	0.95	0.48
11.00	0.59	0.96	0.55	23.00	0.46	0.84	0.30	22.00	0.55	0.95	0.50
10.00	0.64	0.92	0.56	22.00	0.49	0.84	0.33	20.00	0.65	0.95	0.60
9.00	0.71	0.92	0.63	20.00	0.60	0.84	0.44	19.00	0.69	0.90	0.59
8.00	0.75	0.80	0.55	19.00	0.63	0.80	0.43	18.00	0.80	0.85	0.65
7.00	0.81	0.80	0.61	18.00	0.74	0.76	0.50	17.00	0.84	0.85	0.69
6.00	0.86	0.80	0.66	17.00	0.77	0.76	0.53	16.00	0.88	0.85	0.73
5.00	0.88	0.72	0.60	16.00	0.81	0.68	0.49	15.00	0.94	0.85	0.79
4.00	0.90	0.72	0.62	15.00	0.88	0.68	0.56	14.00	0.96	0.75	0.71
3.00	0.92	0.56	0.48	14.00	0.91	0.60	0.51	13.00	0.98	0.70	0.68
2.00	0.93	0.40	0.33	13.00	0.95	0.56	0.51	12.00	1.00	0.60	0.60
1.00	0.95	0.08	0.03	12.00	0.96	0.48	0.44	11.00	1.00	0.40	0.40
0.00	1.00	0.00	0.00	11.00	0.98	0.32	0.30	10.00	1.00	0.30	0.30
				10.00	0.98	0.24	0.22	9.00	1.00	0.20	0.20
				9.00	1.00	0.16	0.16	7.00	1.00	0.15	0.15
				7.00	1.00	0.12	0.12	6.00	1.00	0.10	0.10
				6.00	1.00	0.08	0.08	5.00	1.00	0.00	0.00
				5.00	1.00	0.00	0.00				

TSG, trans-stenotic gradient; SSS, superior sagittal sinus.

## Discussion

In this retrospective study, we evaluated the diagnostic accuracy of venous manometry for identifying elevated ICP in patients with venous sinus stenosis. The objective was to assess how well venous manometry predicts elevated CSF-OP, rather than to establish physiological equivalence between venous pressures and LP measurements. Both SSS pressure and TSG demonstrated strong diagnostic performance. Although the Dandy criteria for IIH require a CSF-OP of 25 cmH₂O, we used 20 cmH₂O as the reference threshold, reflecting the high-normal limit generally considered elevated (15, 16). Notably, combining a TSG ≥ 6 mmHg with an SSS pressure ≥15 mmHg provided the best diagnostic accuracy, yielding 94% sensitivity and 85% specificity for detecting ICP ≥ 20 cmH₂O. Moreover, a TSG ≥ 15 mmHg or an SSS pressure ≥27 mmHg was 100% specific for elevated ICP. These findings suggest that LP may be unnecessary in a subset of patients, potentially sparing them the discomfort and risks of the procedure, particularly in the setting of raised ICP. In our experience, patients often refuse LP, and manometry may be a more acceptable alternative. Although absolute venous pressure measurements can be influenced by factors such as catheter selection (17), sedation, or anesthesia (18), our results indicate that combining SSS pressure with TSG may mitigate these limitations. This represents one of the largest series to date in which venous pressures and LP were obtained during the same procedure, highlighting the diagnostic value of TSG. Importantly, not all patients in this cohort carried a formal diagnosis of IIH at the time of evaluation; some were referred for assessment of possible IIH based on symptoms and venous sinus stenosis identified on MR venography. This explains why a subset of patients demonstrated normal CSF-OP despite undergoing manometry and LP.

Recent studies have demonstrated that intracranial and venous sinus pressures vary with body and head positioning, reflecting the dynamic physiology of CSF and venous outflow (19–21). In our protocol, venous manometry was performed with the patient supine and the head maintained in a straight position, whereas LP opening pressure was measured in the lateral decubitus position. These differing positions can introduce expected physiologic variation in absolute pressure measurements and likely contribute to some of the within-patient differences observed between SSS pressure and CSF-OP. Importantly, despite these positional effects, the predictive relationship between elevated SSS pressure, TSG, and elevated CSF-OP remained strong, suggesting that posture-related shifts influence baseline values more than the diagnostic utility of venous manometry.

Our results confirm a strong correlation between CVP and CSF-OP, reinforcing the hypothesis that venous sinus stenosis contributes to elevated ICP in patients with IIH. This is consistent with prior studies, such as Lee et al., who reported a significant relationship between venous sinus pressures and CSF-OP, with the pressure measurement at the torcula exhibiting the strongest correlation (R^2^ = 0.58) (13). Importantly, this relationship was maintained in patients with both normal and elevated CSF-OP (≥20 cmH_2_O). Our reported R^2^ value for SSS pressures was nearly identical to that reported by Lee et al. Similarly, Iyer et al. reported a strong linear relationship between venous sinus pressures and CSF-OP in patients who had not undergone VSS (11). Previous studies further support our findings regarding the correlation between SSS pressures and CSF-OP (12, 22).

Although classical CSF–venous physiology suggests that SSS pressure is directionally coupled to ICP—and therefore associated with CSF-OP measured on LP—it does not imply numerical equivalence or a uniform identity relationship between these measurements. Consistent with this, our paired analysis demonstrated substantial inter-individual variability between CSF-OP and SSS pressure. This divergence is physiologically expected and likely reflects the influence of venous sinus stenosis, venous compliance, central venous pressure, and intracranial–spinal CSF gradients—all of which can introduce systematic offsets and disrupt a strict 1:1 relationship at the individual level. Additionally, our data reinforce the strong relationship between TSG and CSF-OP. Fargen et al. identified a similar relationship between elevated TSG and increased CSF-OP (11–14). This consistent correlation of CSF-OP with venous pressures and pressure gradients supports the underlying pathophysiological feedback loop in IIH and highlights the role of VSS in reducing these pressures. Furthermore, the degree of CSF-OP, pre-stent TSG, and change in TSG after stenting have been shown to influence outcomes after VSS. McDougall et al. reported a significant correlation between change in TSG and clinical improvement following venous sinus stenting (23).

Elevated CSF-OP can lead to extrinsic venous stenosis and venous congestion, which may drive the TSG. However, venous sinus stenosis is not the only factor contributing to elevated ICP in all patients. A closer look at the outliers in our results revealed that some patients had high CSF-OP without a significant venous pressure gradient. Conversely, others showed a high gradient but normal CSF-OP. Specifically, five patients had a TSG of 8 mmHg or greater with a CSF-OP below 20 cmH_2_O, and three patients had a CSF-OP exceeding 25 cmH_2_O with a TSG below eight mmHg. These findings support the subclassification proposed by Fargen, which suggests that some patients may develop IIH due to elevated systemic CVP rather than pathologic venous stenosis, or from extracranial venous outflow impairment such as Internal Jugular Venous Stenosis (24). The dynamic relationship between CSF pressure, SSS pressure, and CVP was explored by Lalou et al., who showed that SSS pressure and CSF pressure were closely linked during thecal sac saline infusion, except when CSF pressure dropped to a level equal to jugular venous pressure (14). SSS pressure did not decrease below a threshold set by jugular venous pressure, indicating that if CVP rises enough to reduce the typical 3–5 mmHg pressure gradient necessary for CSF reabsorption, ICP may increase. Understanding this subgroup of patients can help interventionalists identify individuals who may not be optimal candidates for stenting. Kahan et al. did not find a relationship between treatment failure and venous gradient, but they did note that higher baseline CSF-OP was associated with increased risk of failure, implying that elevated baseline CVP may decrease the chances of successful stenting (22). Ultimately, a thorough review of the literature and detailed assessment of these patient subgroups support the classification proposed by Fargen et al. (24). However, further research is necessary to define these subgroups better and determine which patients are most likely to benefit from various treatment options for IIH.

Elevations in baseline CVP, often due to conditions such as heart failure or elevated intrathoracic pressure, can lead to increased CSF-OP and symptomatic IIH. In these patients, a high-pressure equilibrium may develop because of the “resistor” function of the venous sinuses and bridging cortical veins. This can lead to venous sinus stenosis through feed-forward mechanisms of extramural compression of the sinuses. While VSS may alleviate symptoms in some cases, this patient group is at increased risk of treatment failure and symptom recurrence due to the underlying pathophysiology. In patients with inadequate venous collateral systems, there is a predisposition to sinus collapse, where even a mild degree of stenosis can result in a significant pressure gradient (25). Additionally, in individuals who have previously undergone VSS, the venous sinuses may no longer exhibit a tight relationship with CSF-OP, suggesting a disruption in the Starling-like resistor mechanism that the venous sinuses normally exhibit. This highlights that VSS is not a panacea for all patients with IIH. While it may be curative in some cases, it may be highly effective but not curative in others, and entirely ineffective in a subset of patients.

Our study identified highly sensitive and specific cut-off values for predicting elevated CSF-OP using venous manometry, thereby reinforcing the clinical utility of this technique and laying a foundation for future research to refine these thresholds for informed treatment decisions. While LP or an ICP monitor remains the gold standard for assessing ICP, our findings support prior literature and further validate the diagnostic accuracy of venous manometry in predicting elevated CSF-OP. In patients with an elevated TSG and SSS pressure, clinicians can reasonably expect that an LP would reveal elevated CSF-OP. However, there is significant variability among interventionalists in their preferred TSG cut-off for determining eligibility for VSS. Although a TSG cut-off of 8 mmHg is commonly used, this threshold remains somewhat arbitrary. Our study validates alternative cut-off values and directly links them to what is clinically most relevant—the patient’s CSF-OP—thereby improving decision-making in treatment planning. The predictive power of TSG not only enhances clinical decision-making but also raises important questions about the necessity of LP in the VSS work-up. Our results emphasize the need for further research into the predictive value of TSG and the potential for eliminating LP from the pre-VSS evaluation.

This study has several limitations. First, it is a single-center, retrospective review, which may limit the generalizability of the findings. Variability in venous manometry techniques across centers is also a potential issue, as pressure gradients can differ depending on the methodology. However, Fargen et al. demonstrated that correcting for various physiologic factors can standardize these pressure gradients across different settings. In particular, without strict anesthetic and physiologic considerations, pressures may vary between awake and general anesthesia conditions. In our cohort, all measurements were assessed with local anesthetic, using the same large-bore microcatheter (0.027 inches) for manometry and the same 22G needle for LP, with the patient in the left lateral decubitus position. A large-bore microcatheter is essential for accurate manometry.

Furthermore, the purpose of this study was to assess the diagnostic accuracy of venous manometry in predicting elevated CSF-OP. It does not identify ideal candidates for VSS, which would require a more comprehensive analysis of post-VSS outcomes in relation to preoperative characteristics, including manometry measurements and CSF-OP. This series may also be subject to selection bias, as this study represents a group of patients with a tentative diagnosis of IIH and venous sinus stenosis who the senior author considered good candidates for manometry and LP. A broader sample may yield more generalizable results.

## Conclusion

Venous manometry accurately predicts elevated ICP in patients with venous sinus stenosis. SSS pressure and TSG strongly correlated with CSF-OP, with thresholds of SSS ≥ 15 mmHg and TSG ≥ 6 mmHg providing excellent diagnostic performance. The combination of these parameters achieved the highest accuracy, supporting venous manometry as a reliable surrogate for LP in identifying intracranial hypertension and guiding patient selection for VSS. Prospective, multicenter validation of these thresholds is warranted to standardize their use in the diagnostic evaluation of idiopathic intracranial hypertension.

## Data Availability

The original contributions presented in the study are included in the article/supplementary material, further inquiries can be directed to the corresponding authors.
